# Quantitative Changes and Transformation Mechanisms of Saponin Components in Chinese Herbal Medicines during Storage and Processing: A Review

**DOI:** 10.3390/molecules29184486

**Published:** 2024-09-21

**Authors:** Yuhang Wu, Hui Zheng, Tao Zheng, Jiani Jiang, Yao Xu, Fan Jia, Kai He, Yong Yang

**Affiliations:** 1 School of Pharmacy, Hunan University of Chinese Medicine, Changsha 410000, China; 15387030919@163.com (Y.W.); huizheng0104@163.com (H.Z.); 004247@hnucm.edu.cn (T.Z.); jiangjiani0320@163.com (J.J.); 20223684@stu.hnucm.edu.cn (Y.X.); jiafan1227@163.com (F.J.); 2 School of Pharmaceutical Science, Hunan University of Medicine, Huaihua 418000, China

**Keywords:** Chinese herbal medicines, saponin components, storage and processing, mechanism

## Abstract

Saponins are an important class of active components in Chinese herbal medicines (CHMs), which are present in large quantities in *Ginseng Radix et Rhizoma*, *Notoginseng Radix et Rhizoma*, *Polygonati Rhizoma*, etc., and have immune regulation, anti-tumor, anti-inflammatory, anti-cardiovascular disease, and hypoglycemic activities. Storage and processing are essential processes in the production process of CHMs which affect the stability of saponin components and then reduce the medicinal and economic value. Therefore, it is of great importance to investigate the effects of storage and processing conditions on the content of saponin components in CHMs. In this paper, the effects of various storage and processing factors, including temperature, pH, enzymes, meta lions, extraction methods, etc., on the saponin content of CHMs are investigated and the underlying mechanisms for the quantitative changes of saponin are summarized. These findings may provide technical guidance for the production and processing of saponin-rich CHMs.

## 1. Introduction

**Figure 1:**
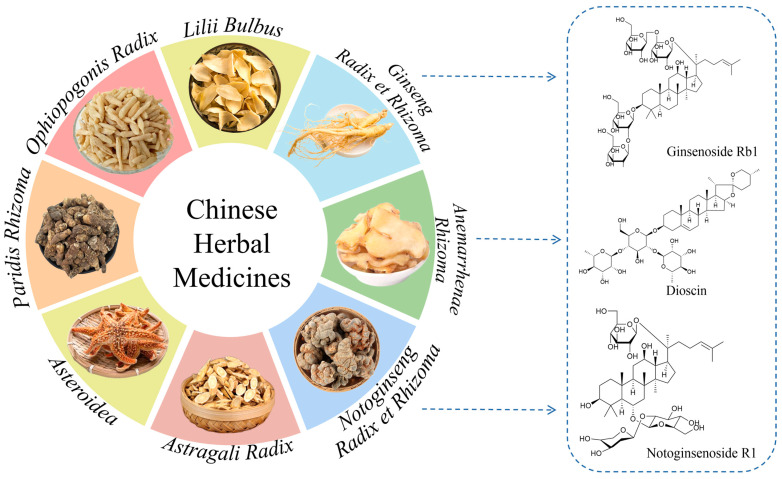
Some Chinese herbal medicines (CHMs) and their saponin components.

Saponin components are widely found in natural products and a large number of Chinese herbal medicines (CHMs) resources such as *Ginseng Radix et Rhizoma*, *Notoginseng Radix et Rhizoma*, *Polygonati Rhizoma*, *Paridis Rhizoma*, and *Asteroidea* (Figure 1) [1,2,3]. Saponins are glycosidic compounds consisting of triterpenoids or spirostane compounds combined with glycosyl group through glycosidic bonds. According to their sapogenins, they can be divided into triterpenoid saponins and steroidal saponins. Triterpenoid saponins and steroidal saponins are derived from the mevalonic acid pathway. The fundamental structure of triterpenoid saponins is composed of six isoprenoid structural units with 30 carbons, which are primarily classified as either tetracyclic or pentacyclic triterpenoids [4]. Steroidal sapogenins, which are spirostanoid derivatives, feature six-membered ring structures with 27 carbons and are mainly classified into spirostanol, isospirostanol, furostanol, and pseudospirostanol [5]. Contemporary research has identified numerous saponins with pharmacological activities such as immune modulation, anti-tumor activity, anti-inflammatory effects, antidepressant actions, antimicrobial properties, and regulation of cardiovascular and cerebrovascular functions, as well as hypoglycemic effects [6,7,8]. The number, position, and configuration of the glycosides, as well as the structure of the sapogenins, are closely related to their bio-activities [9,10]. In general, the number of glycosyl groups in ginsenosides is inversely correlated with their anti-tumor activity, indicating that an increased number of sugar moieties is linked to diminished anti-cancer efficacy [11]. The glycosylation of steroidal saponins at the C-3 hydroxyl position is beneficial for enhancing their anti-diabetic activity. 20 (S)-Rg3 and 20 (R)-Rg3 are isomers of each other, but 20 (S)-Rg3-OH is geometrically close to the C-12 hydroxyl group of ginsenosides. 20 (R)-Rg3-OH is far from the C-12 hydroxyl group. This results in the superior ability of 20 (S)-Rg3 to scavenge hydroxyl radicals compared to 20 (R)-Rg3 [12].

Saponins are water-soluble glycosides whose structure and physicochemical properties are influenced by various factors. As the primary active components in many CHMs, understanding the changes in their content and the underlying mechanisms during the storage and processing of CHMs is crucial for ensuring quality control in CHMs production. Storage and processing are essential steps in the journey from raw material to the final product, and from the origin to the consumer. Consequently, the storage and processing conditions significantly affect the saponin contents and subsequently impact the clinical efficacy. To gain a deeper understanding of saponins as active components in CHMs and to elucidate the patterns and quantitative mechanisms of their content changes during storage and processing, this paper reviews the impact of storage and processing conditions on saponin contents in CHMs. Furthermore, it discusses the main influencing mechanisms to provide technical guidance for the production of saponin-rich CHMs.

## 2. Effect of Storage Condition on the Saponin Components of CHMs

Storage is the process of transferring CHMs from the production end to the consumption end. The stability of active ingredients in CHMs can be significantly affected by storage conditions and methods, which may result in alterations to their composition and content. The stability of chemical components, including saponins, in CHMs can be influenced by a number of factors, including storage temperature, humidity, light, oxygen content, packaging, and others. These factors can impact the appearance and medicinal qualities of the CHMs.

### 2.1. Storage Temperature

In general, high temperatures can accelerate chemical reactions, which may result in the degradation and transformation of saponin components. After a four-week storage period under light-protected conditions, significant variations in the total saponin contents of *Polygonatum Cyrtonema Hua* were observed across different temperatures. The samples stored at −20 °C exhibited the highest saponin contents, followed by those stored at 4 °C, and the samples stored at room temperature showed the lowest saponin contents [13]. Consequently, low temperatures are conducive to the reduction in saponin degradation during the storage period. The contents of macranthoidin B and dipsacoside B in *Lonicera Macranthoides Hand.-Mazz.* were found to decrease as the storage temperature increased [14]. After storing *Dioscorea Zingiberensis C. H. Wright* (DZW) tubers at 4 °C and −20 °C for 30 days, it was observed that the water-soluble saponin contents of DZW tubers decreased by 42.5% at 4 °C while remaining almost unchanged at −20 °C [15]. After adjusting pH, *Ginseng Radix et Rhizoma Rubra* extract was subjected to thermal treatment at 105 °C for 5 min and then stored at 5 °C, 25 °C, and 45 °C for 11 weeks. This study revealed that the levels of ginsenosides Rg1, Rb1, and Rh1 decreased progressively over the storage period, with higher storage temperatures correlating to a more pronounced reduction in their content, but the content of ginsenoside Rg3 exhibited an increase post-storage, and this increase was positively associated with the elevation of storage temperature [16]. The study concludes that the observed increase in ginsenoside Rg3 content is likely due to the transformation of other saponins under high-temperature conditions [17]. For example, increasing the temperature can cause the removal of two glucose molecules from ginsenoside Rb1 to form Rg3. Similarly, the temperature-dependent removal of arabinose and glucose from Rb2 may lead to the production of Rg3. Additionally, one molecule each of xylose and glucose can be removed from Rb3 to yield Rg3 [18,19]. The effect of storage temperature on the saponin components within CHMs is significant. It is therefore recommended that saponin-rich CHMs be stored at low temperatures whenever feasible.

### 2.2. Storage Humidity

CHMs is susceptible to moisture absorption and deliquescence when exposed to high-humidity conditions during the storage period. Such high-humidity conditions not only present a risk of mold and pest infestation, but also accelerate the degradation of saponin components. After a 21-day storage period, the total saponin content of dehydrated *Panacis Quinquefolii Radix* exhibited a significant reduction in the high-humidity environment (80~90% RH), a slight reduction in the medium-humidity environment (40~50% RH), and a minimal change in the low-humidity environment (20~30% RH). Consequently, high-humidity environments were found to accelerate the degradation of saponin components [20]. After four months of storage, the total saponin content in *Codonopsis Radix* slices with varying moisture levels was assessed under different conditions, including room temperature, cool storage, refrigeration, accelerated conditions (40 ± 2 °C, 75 ± 5% relative humidity, normobaric oxygen), and low-oxygen curing. The findings indicate that the slices exhibited the lowest reduction in total saponin content in low-temperature, low-humidity, and low-oxygen environments. In contrast, storage at room temperature led to a more significant degradation, indicating that a cold, dry, and oxygen-limited environment is optimal for preserving the quality of *Codonopsis Radix*. Furthermore, storage humidity significantly influences the degradation rate of saponins. When the relative humidity exceeds 75% and nutritional conditions are favorable, molds can proliferate rapidly [21]. Therefore, it is recommended that CHMs containing saponins as their primary components be stored in low-humidity environments whenever possible, given the demonstrated benefits for preserving saponin content and quality.

### 2.3. Light Exposure

Exposure to light can trigger the degradation of photolabile compounds, such as phenols, volatile oils, pigments, and flavonoids, which are commonly found in CHMs. This photodegradation not only affects the stability of these compounds by altering their chemical structures, but also potentially reduces their medicinal efficacy. Moreover, the presence of unsaturated bonds in some components makes them particularly susceptible to certain photochemical reactions, including photocycloaddition reactions. Two portions of the saponin extract from *Star Anise* were kept in the sun and shade for 3 h, respectively, after which their saponin contents were tested. The results indicated that, under the experimental conditions, there was no statistically significant change in saponin contents in the dark storage group. In contrast, exposure to sunlight resulted in a decrease in saponin contents, suggesting that light irradiation may induce photodegradation of these components [22]. A study on *Polygonatum Cyrtonema Hua* demonstrated that after 4 weeks of storage, the total saponin contents decreased by 39.1% in the dark and by 43.4% in the light. These results suggest that the degradation and loss of total saponins in *Polygonatum cyrtonema Hua* can be mitigated by light-sheltered storage conditions compared to light-exposed storage conditions [13]. The irradiation of three types of panax notoginseng saponins at a light intensity of 4500 ± 500 lx for a period of 10 days resulted in a more than 5% higher loss of total saponins in *Notoginseng radix et rhizoma* compared to the unirradiated control group [23]. Saponins generally exhibit stronger light stability compared to phenols, volatile oils, and pigments. However, light irradiation can still lead to storage loss of saponins, especially during long-term storage. Therefore, light-proof storage is recommended for saponin-containing CHMs and their products.

### 2.4. Oxygen and Packaging

Oxygen in the storage environment is a critical factor that can significantly affect the stability of the chemical constituents within CHMs. The choice of storage and packaging methods can significantly dictate the degree of oxygen exposure, which in turn influences the preservation state of CHMs. A study on the impact of various packaging methods on the stability of macranthoidin B and dipsacoside B in *Lonicera Macranthoides Hand.-Mazz.* revealed that the storage retention rates of these two saponins were highest in plastic bags subjected to vacuum sealing, followed by non-vacuumized plastic bags, woven bags with inner film sealing, woven bags, paper bags, and hemp bags. The vacuum-sealed packaging demonstrated the best preservation of saponin stability, suggesting that sealing and oxygen barrier properties are effective strategies for maintaining the integrity of saponins [14]. The study investigated the effects of different packaging materials on the saponin content of *Bacopa Monnieri* (L.) Wettst. after storage and found that packaging with 125 μm high-density polyethylene, which offered superior sealing performance, was most effective in reducing the degradation of saponins. This method demonstrated a significant advantage over other packaging materials, including 25 μm polypropylene-lined jute bags, woven bags, and 5-layer corrugated fiberboard boxes, in preserving the saponin content of *Bacopa monnieri* (L.) Wettst [24]. The implementation of effective packaging techniques can markedly diminish or potentially eliminate the exposure of CHMs to atmospheric air, particularly oxygen. It is therefore acknowledged that the utilization of sealed or vacuum packaging for saponin-rich CHMs represents an efficacious and recommended strategy for the preservation of their stability and bioactivity.

## 3. Effect of Processing Technology on the Saponin Components of CHMs

During the transformation of CHMs into downstream products, the active constituents are susceptible to the effects of processing conditions, which can result in either losses or improvements in processing efficiency. Different processing techniques such as drying, pulverization, and extraction, as well as conditions such as temperature, pH, enzymes, and the presence of metal ions, have a significant impact on the quality attributes of CHMs, including the saponin contents.

### 3.1. Effect of Processing Methods on the Saponin Components of CHMs

#### 3.1.1. Drying

Drying is the most important post-harvest treatment for CHMs. It improves their storage properties, minimizes degradation, and facilitates transport [25]. Traditional drying methods include shade drying, sun drying, and oven drying, while modern techniques include hot air drying, microwave drying, infrared drying, and freeze drying [26,27].

1. Traditional shade drying can prevent quality loss due to exposure to high temperatures, but glycoside hydrolases in CHMs, which remain highly active, can hydrolyze saponins to secondary saponins or sapogenins [28]. The shade-drying method is an inefficient process and is rarely used today. CHMs with saponins as markers or functional components are not suitable for this method [29]. Sun drying is highly dependent on natural climatic conditions, resulting in unstable drying quality. Consequently, it has largely been replaced by the oven-drying method in current production practices [30]. In the oven-drying method, the drying temperature significantly affects the saponin components of *Notoginseng Radix et Rhizoma*. After fresh cutting, the samples were dried at temperatures of 40 °C, 50 °C, and 60 °C. The contents of saponins R1, Rg1, and Rb1 were observed to initially increase and then decrease with the increasing temperature, peaking at 50 °C [31]. Fresh *Ginseng Radix et Rhizoma* was subjected to drying at temperatures of 30 °C, 40 °C, 50 °C, 55 °C, and 60 °C. The contents of ginsenosides Rg1 and Re initially increased with the rise in temperature and then decreased, reaching their peak at 50 °C [32]. High drying temperatures can transform the saponins contained in some medicinal materials into rarer saponins. Ginsenosides may hydrolyze and lose part of their sugar groups at high temperatures, turning into less polar saponins, which are more easily absorbed into the blood circulation [33,34,35]. Malonyl ginsenosides in ginseng are prone to decarboxylation and subsequent degradation at elevated temperatures, transforming into other neutral saponins and rare saponins [36,37,38]. Although the low-temperature drying method cannot completely inhibit enzyme activity, which may lead to the hydrolysis of some saponins, it results in a significantly better retention rate of total saponin contents compared to the sun-drying method. High-temperature drying effectively inactivates endogenous enzymes, but also predisposes saponin components to decomposition or conversion [30].
2. In hot air drying, heated air is employed as the drying medium, transferring heat from the surface to the interior of CHMs through a temperature gradient, thereby achieving the objective of drying. A study investigated the effects of boiling, hot air drying, sun drying, and shade drying on the levels of ginsenoside Ro and chikusetsu saponin IVa in *Panacis Majoris Rhizoma*, and the results indicated that the saponin contents was higher in samples subjected to hot air drying than in those subjected to sun drying, shade drying, or boiling [39]. The total saponin contents of *Polygonati Rhizoma* dried by hot air was higher than that obtained by the sun-drying method [40]. The total saponin content of *Ginseng Radix et Rhizoma* dried in hot air at a low temperature of 40 to 50 °C was higher than that of samples dried by the sun-drying, shade-drying, and microwave-drying methods [41]. Hot air drying has several advantages over shade drying and sun drying, including reduced drying time, improved efficiency, and increased saponin yield. However, it can also cause crusting on the surface of the herb, which can impede the diffusion of internal moisture. Therefore, when employing hot air drying, it is crucial to control the pile thickness of the material to prevent incomplete drying or excessively long drying times [42].
3. Microwave drying utilizes microwave energy to rapidly heat CHMs from the inside out, enabling efficient and thorough drying. A comparison of five drying methods on the total saponin contents of *Polygonati Rhizoma* revealed the following order of efficacy: atmospheric microwave drying, vacuum microwave drying, vacuum drying, hot air drying, and natural drying. The total saponin contents of the samples dried using both microwave methods were close to the maximum value recorded in the study [40]. However, some studies have reported that the total saponin contents of microwave-dried *Paridis Rhizoma* were close to or lower than those of samples dried using low-temperature methods [43]. The contents of 14 ginsenosides, including Rg1, Re, Rb1, Rc, Rb2, Rb3, Rd, and their malonyl ginsenosides, in samples of *Ginseng Radix et Rhizoma* and *Panacis Quinquefolii Radix* flowers dried using microwave energy were all found to be lower than those in samples dried at 40 °C. This reduction may be due to the thermal sensitivity of certain ginsenosides, which can be destroyed by the localized high heat generated during microwave drying [44]. Microwave drying has been shown to yield higher amounts of saponins in some CHMs compared to natural drying. However, controlling the local temperature during microwave drying is challenging. Therefore, further in-depth research is needed to effectively apply microwave-drying technology to the processing of saponin-rich CHMs.
4. Infrared drying utilizes infrared radiation to directly heat CHM, causing the material to heat up internally. This internal heat then facilitates the migration of moisture through heat conduction, thereby achieving the drying objective. Infrared radiation is a type of electromagnetic wave with wavelengths ranging from 0.76 to 1000 μm. It is categorized into near, middle, and far infrared based on these wavelengths [45]. A comparison of the total saponin contents in *Ginseng Radix et Rhizoma* under infrared drying, hot air drying, and combined infrared–hot air drying conditions at temperatures of 50 °C, 60 °C, and 70 °C showed that the saponin contents were higher in samples dried by the infrared and combined infrared and hot air methods than those dried by hot air alone at the same temperature [46]. A comparative study of sun-drying, shade-drying, oven-drying, far-infrared-drying, and microwave-drying methods on *Anemarrhenae Rhizoma* revealed that far-infrared drying preserved the highest content of timosaponin A-III [47]. The above study discovered that infrared drying can increase the content of saponin components in CHMs, such as *Panax ginseng* and *Anemarrhena asphodeloides rhizoma*.
5. Freeze drying, or lyophilization, effectively reduces changes in the chemical composition and preserves the appearance of natural raw materials [39,48]. One study investigated and compared the effects of various drying methods, including shade drying, sun drying, freeze drying, microwave drying, and hot air drying (at temperatures ranging from 40 to 80 °C), on the total saponin contents of *Ginseng Radix et Rhizoma* pieces. The results indicated that freeze-dried samples had the highest total saponin contents, followed by those dried using low-temperature (40 to 50 °C) hot air drying. Shade drying and other mild drying methods yielded intermediate contents, while microwave drying resulted in the lowest saponin content [41]. The study showed that the total saponin contents of freeze-dried *Ginseng Radix et Rhizoma* were significantly higher than those of samples dried by the hot air- and vacuum microwave-drying methods, indicating the superiority of freeze drying in preserving saponin contents [49]. Freeze drying is recognized as an industrialized drying technology that excels in preserving the fresh quality of raw materials. However, its application in the processing of common CHMs is limited due to the high energy costs associated with the process.

The characteristics and principles of the various drying methods differ significantly. Since the saponin components and physicochemical stability of different CHMs also vary, the choice of drying method in industrial production should balance drying efficiency and quality to achieve optimal results.

#### 3.1.2. Pulverization

Pulverization is a routine process in the production and processing of CHMs. Traditional methods such as “water flying”, “pounding”, and “filing” are mainly used for the preparation of mineral medicines, precious herbs, animal-derived medicines, and certain special CHMs [50]. Pulverization increases the surface area and porosity of CHMs raw materials, thereby enhancing the dissolution and absorption of their active ingredients [51]. The dissolution of total saponins in *Notoginseng Radix et Rhizoma* powder was investigated over a range of median particle sizes: 18.01, 15.76, 10.21, 8.17, 6.16, 5.61, 5.29, 4.94, 4.80, 4.24, 3.76, 3.30, and 2.53 μm. The study found that total saponin dissolution initially increased with decreasing particle size and then decreased. The maximum saponin dissolution was observed at a median particle size of 4.24 μm [52]. The study on *Ginseng Radix et Rhizoma* observed a similar trend in saponin dissolution rate, which increased and then decreased with the decrease in the particle size of the powdered material. The highest saponin dissolution rate was achieved when the particle size was in the range of 0.5 to 1 μm [53]. Particle size is a critical parameter in the pulverization process of CHMs. The reduction in the saponin extraction rate for some CHMs may be attributed to the high-temperature degradation caused by pulverization and the formation of agglomerates in ultrafine powders, which can hinder the extraction efficiency. The smaller the particle size of the pulverized herbs, the longer the pulverization time and the greater the resulting temperature rise. This increase in temperature can lead to pyrolysis of the saponins. At the same time, the formation of agglomerates between CHMs particles in pulverized powders reduces the effective contact area with solvents, thereby inhibiting dissolution.

#### 3.1.3. Extraction

The choice of extraction method has a significant influence on the extraction rate of active ingredients or bioactive components in CHMs. An appropriate extraction method should take into account not only the physical and chemical properties of the raw materials and the components to be extracted, but also the economic viability of the process. Currently, several extraction methods are commonly used in the production of CHMs, including maceration, reflux, ultrasound-assisted, microwave-assisted, enzyme-assisted, and supercritical fluid extraction [54,55,56].

1. The ultrasonic-assisted extraction method enhances the solvent extraction process by incorporating ultrasonic energy during the extraction. Ultrasound-assisted extraction is widely utilized in the field of phytochemical analysis due to its high efficiency, energy-saving properties, and cost-effectiveness. One study investigated the effects of decoction, reflux extraction, and ultrasonic-assisted extraction on *Notoginseng Radix et Rhizoma* using panax notoginseng saponins, ginsenosides Rb1 and Rg1, as indices. The results indicated that ultrasonic-assisted extraction yielded the highest saponin extraction efficiency [57]. Ultrasonic-assisted extraction of saponins from *Chenopodium Qquinoa Willd.* seeds demonstrated a significant advantage over the reflux extraction method, with a higher saponin extraction rate [58]. The extraction rate of jujube saponin A from *Ziziphi Spinosae* seeds was found to be 14.25% higher compared to reflux extraction at 65 °C [59]. Ultrasonic-assisted extraction accelerates the transfer of saponins into the solvent and enhances their dissolution through the cavitation, mechanical, and thermal effects generated by ultrasound [60,61].
2. The microwave-assisted extraction method combines microwave energy with conventional extraction techniques, utilizing the thermal effects of microwaves to extract the active components from CHMs. Methanol and ethanol were used as extraction solvents to compare the saponin constituents of Chenopodium album via microwave-assisted extraction and soxhlet extraction. The yields from microwave-assisted extraction were 3.05 ± 0.112 mg/g for methanol and 3.22 ± 0.061 mg/g for ethanol, which were 68.5% and 75.0% higher, respectively, than those obtained by soxhlet extraction [62]. A comparison of microwave-assisted extraction, solvent extraction, and ultrasonic-assisted extraction for total saponins in *Asteroidea* revealed that microwave-assisted extraction yielded an extraction rate of 60.3 ± 0.6 mg/g. This rate was 3.3 times higher than that of ultrasonic-assisted extraction and 7.8 times higher than the solvent extraction method [63]. Microwave-assisted extraction leverages microwave energy to rupture cell walls and membranes, accelerating the release of saponin components and thereby enhancing dissolution efficiency. However, temperature control during microwave heating is a challenge with this extraction method [64].
3. Enzyme-assisted extraction involves the use of exogenous enzymes to break down the cell wall structure, thereby allowing the active ingredients in CHMs to be fully released into the extraction medium. A comparison of the ethanol reflux method, cellulase-assisted extraction, pectinase-assisted extraction, and the combined cellulase–pectinase-assisted extraction on *Gynostemma pentaphyllum (Thunb.) Makino* revealed that the gypenoside extraction rates followed this order: combined cellulase–pectinase-assisted extraction > cellulase-assisted extraction > pectinase-assisted extraction > ethanol reflux extraction. Notably, the extraction rate of gypenosides by the combined cellulase–pectinase method was 31% higher than that of the ethanol reflux extraction method [65]. Enzymatic extraction methods yielded higher saponin contents from *Chenopodium quinoa Willd.* than the ethanol reflux extraction method. Specifically, the cellulase–pectinase-assisted extraction yielded a saponin content of 42.220 mg/g, which was 15.935 mg/g higher than ethanol extraction, 8.531 mg/g higher than pectinase extraction, and 6.647 mg/g higher than cellulase extraction alone [66]. Cellulase and pectinase effectively degrade pectin and cellulose in plant cell walls and intercellular substances, disrupting the cell wall structure of plant tissues. This action reduces cell wall density, which facilitates the dissolution of saponin components. However, enzymatic extraction requires precise experimental conditions; an inappropriate environment can easily lead to enzyme inactivation and denaturation.
4. Supercritical fluid extraction utilizes the dissolution properties of supercritical fluids, which change with variations in density. By adjusting the pressure or temperature, the density of the supercritical fluid can be significantly altered, thereby modifying its dissolution capabilities. Water reflux extraction, ethanol reflux extraction, enzymatic extraction, percolation extraction, and supercritical CO_2_ fluid extraction were employed to extract total saponins from *Smilacis Chinae Rhizoma*. Among these methods, supercritical CO_2_ fluid extraction yielded the highest total saponin content, which was 3.39, 1.34, 1.36, and 1.47 times higher than those obtained by water reflux extraction, ethanol reflux extraction, enzymatic extraction, and percolation extraction, respectively [67]. A comparison of the saponin contents in *Astragalus Radix* extracted by water reflux extraction, ethanol reflux extraction, and supercritical fluid extraction revealed that the supercritical fluid extraction method yielded the highest extraction rates for total saponins and astragaloside A, with values of 1242.5 ± 18.9 μg/g and 1211.3 ± 16.9 μg/g, respectively [68]. Supercritical fluid extraction can selectively extract a variety of compounds by adjusting pressure or temperature. However, the extraction of high-molecular-weight and polar saponins often requires the use of entrainment agents. The inclusion of these agents can further complicate the high-pressure phase equilibrium, leading to variable pressure operation, increased costs, and challenges in scaling up for industrial production [69].

Table 1 summarizes the advantages and disadvantages of various extraction methods for saponins from CHMs. The extraction process for CHMs should be tailored to the specific organizational and saponin compositions in order to select the most effective extraction method, thereby maximizing the extraction rate. A combination of methods can also be employed to increase the saponin yield, such as ultrasonic- and microwave-assisted hydro-distillation [70], ultrasonic-assisted enzymatic extraction [71], ultrasound-assisted extraction with natural deep eutectic solvent [72], etc.

### 3.2. Effect of Processing Conditions on the Saponin Components of CHMs

#### 3.2.1. Processing Temperature

The rate of chemical reactions and the molecular diffusion of substances is closely related to temperature. Therefore, the processing temperature of saponin-containing CHMs has two significant effects: firstly, an increase in temperature can enhance the diffusion and solubility of saponins, which is beneficial for extraction. Conversely, high temperatures can lead to the pyrolytic degradation of the saponins, which is detrimental to maintaining their integrity. When total saponins were extracted from *Ginseng Radix et Rhizoma* adventitious roots using the reflux method, it was observed that the total saponin content initially increased and then decreased as the extraction temperature was raised from 40 °C to 90 °C, with the peak content occurring at 70 °C [78]. A study investigating the effect of combined microwave and infrared drying on white ginseng slices revealed that as the far-infrared temperature increased from 50 °C to 65 °C, the ginsenoside content initially increased and then decreased, reaching a peak at 55 °C [34]. When ultrasound-assisted extraction was applied to *Polygonati Rhizoma*, it was observed that the extraction rate initially increased and then decreased as the extraction temperature was raised from 30 °C to 70 °C, with the peak extraction rate occurring at 60 °C [79]. High-temperature steaming of fresh ginseng during the process of transforming it into red ginseng can induce demalonylation, deglycosylation, and dehydration reactions of saponins. Consequently, red ginseng steamed at lower temperatures has a higher content of polar ginsenosides, whereas red ginseng steamed at higher temperatures contains a higher amount of less polar ginsenosides [80]. Malonyl ginsenosides are susceptible to hydrolysis at high temperatures, which removes the malonyl group and results in the formation of other neutral saponins as well as the formation of rarer saponin [36,37,44]. CHMs processed at moderate temperatures (50~70 °C) show improved solubilization of saponin components. However, at higher temperatures, the chemical bonds of saponins may be disrupted, leading to a reorganization that affects their solubility and can convert them into other substances. Therefore, control of the processing temperature is critical for CHMs containing saponins.

#### 3.2.2. Conditional pH

The glycosidic bonds within the saponin components are sensitive to pH levels, and saponins are readily hydrolyzed to produce secondary saponins or sapogenins when exposed to strong acids or bases. Decoction experiments with saikosaponin A at pH levels of 6.96, 6.21, 5.94, 5.62, and 5.06 revealed that the degradation rate of saikosaponin A increased progressively as the pH decreased within the experimental range. These results indicate that acidic processing conditions may be detrimental to the stability of saikosaponin A [81]. Alkali treatment of the stems and leaves of ginseng significantly increased the ginsenoside Rg2 content, which was found to be 7.5 times higher than that of the untreated control. The results indicated that ginsenoside Re could lose one glucose molecule to form Rg2 under alkaline conditions, resulting in a significant increase in the conversion rate of ginsenoside Rg2 following alkali treatment [82]. Ginsenosides were extracted from *Ginseng Radix et Rhizoma* by refluxing in aqueous solutions with pH values ranging from 2.4 to 11.2. The results showed that the solubility of ginsenosides decreased with the increasing acidity of the solution. Furthermore, it was found that increasing the alkalinity of the solution appropriately increased the dissolution rate of ginsenosides [83]. Reflux extraction of *Bupleurum Radix* in ethanol solutions with pH values ranging from 7 to 11 revealed that the yield of saikosaponin initially increased and then decreased with increasing pH. The highest yield of saikosaponin was achieved at a pH of 8 [84]. During the extraction and processing of CHMs containing saponins, the pH of the extractor can affect the stability of the saponins, potentially leading to hydrolysis of the glycosidic bond and conversion of saponins to sapogenins. This process may result in a reduced saponin extraction rate. Previous studies have shown that increased acidity in the solution is detrimental to saponin stabilization. Therefore, careful selection of the correct pH during the extraction process is essential.

#### 3.2.3. Enzymes

Enzymes are ubiquitous in biological systems, and both the indigenous glycoside hydrolases present in CHMs and the exogenous enzymes added during processing can influence the integrity and transformation of saponins. Enzymes possess specificity; glycosidases are capable of cleaving glycosidic bonds, which results in alterations to saponin contents and structure. Additionally, cellulase, pectinase, and other enzymes that degrade plant cell wall polysaccharides facilitate the release of intracellular saponins, thus modifying their content. A study investigated the relationship between the content of spirostanol saponins and the activity of furostanol glycoside 26-*O*-*β*-glucosidase (F26G) in the roots and stems of dried *Paridis Rhizoma* and found a positive correlation. The activity of F26G in *Paridis Rhizoma* was associated with the conversion of furostanol saponins into spirostanol saponins by cleaving the glycosidic bond at the C-26 position [85]. The F26G present in *Anemarrhenae Rhizoma* is capable of cleaving the glycosidic bond at the C-26 position of furostanoid saponins. This enzymatic activity permits the F ring to undergo a structural transformation, resulting in the synthesis of corresponding spirostanoid-type saponins [86]. During the drying process of *Gynostemma Pentaphyllum (Thunb.) Makino*, gypenoside LVI can be sequentially transformed by the action of glucosidase. Initially, the enzyme removes the lateral glucose at the C-3 position, yielding gypenoside LVII. Subsequently, the medial glucose at the C-3 position is further cleaved, resulting in the formation of gypenoside LXXVII [28]. The conversion rates of rare prosaikogenin A from saikosaponin B1 were evaluated by the hydrolytic action of β-glucosidase, β-glucanase, cellulase, and snailase enzymes. The results showed that snailase, a complex enzyme consisting of more than 20 individual enzymes, significantly outperformed the other three enzymes in terms of conversion rate [87]. The incorporation of complex polysaccharide enzymes and pectinases during the extraction process resulted in a significant increase in the total extracted ginsenoside content, with a 1.23- to 1.43-fold increase observed. In addition, the concentrations of certain ginsenosides, including Rb2, Rc, Rd, Re, Rf, Rg1, and Rg2, were increased by 1.09 to 1.95 times [88]. Saponin extraction from *Chenopodium Quinoa Willd.* using enzymatic methods yielded higher contents than ethanol reflux extraction. Specifically, the total saponin extraction rates followed this order of magnitude: cellulase–pectinase-assisted extraction was the most effective, followed by cellulase-assisted extraction, pectinase-assisted extraction, and ethanol reflux extraction as the least effective method [66]. Therefore, in the processing of CHMs, it is crucial to strategically decide whether to harness or eliminate enzymatic activity to meet the requirements for specific saponin components.

#### 3.2.4. Metal Ions

Recent findings have revealed that metal ions can form specific complexes with saponins, thereby affecting their processing stability. A comparative study evaluated the chelating ability of total saponins extracted from *Chenopodium Quinoa Willd.* against Fe^2+^ in comparison with EDTA. The results indicated that, while the chelating capacity of quinoa total saponins was less pronounced than that of EDTA, both exhibited a positive correlation between chelating capacity and concentration. Specifically, the chelating rate of quinoa total saponins was found to be 61.42% at a concentration of 10 mg/mL [89]. A comparative analysis of the complexation rates of various CHMs decoctions with Fe^3+^ showed that eight CHMs characterized by high concentrations of triterpenoid saponins, including *Dipsaci Radix*, *Cynomorii Herba*, *Morindae Officinalis Radix*, *Astragali Complanati Semen*, *Trigonella foenum-graecum L*, *Drynariae Rhizoma*, *Curculiginis Rhizoma*, and *Cibotii Rhizoma*, exhibited higher complexation rates (ranged from 63.57% to 84.28%) [90]. Metal ions have the capacity to facilitate the conversion of ginsenosides into rare saponins. It has been reported that the metal ion Nb^5+^ catalyzes the conversion of ginsenoside Rb1 into ginsenosides Rg3, Rk1, and Rg5 in anhydrous ethanol solvent [91]. Similarly, Fe^3+^ has been demonstrated to facilitate the formation of ginsenosides Rk1 and Rg5 from ginsenoside Rb1 under identical conditions [92]. Furthermore, Fe^3+^ has been demonstrated to catalyze the cleavage of ginsenoside Rg1 to form Rh1. In addition, dehydration reactions have been observed to lead to the formation of Rk3 and Rh4 [93]. Metal ions can form complexes with saponins or act as catalysts, thereby influencing the structure and composition of these compounds. It is therefore obvious that the role of metal ions in the production and processing of saponin-containing materials should be exploited rationally in order to optimize extraction yields.

## 4. Transformation Mechanisms of Saponins in Storage and Processing of CHMs

The structure and content of saponins in CHMs may be altered by chemical reactions involving heat, acid–base, and enzymes during processing. These reactions cover a range of processes, including dissolution effects, hydrolysis reactions, isomerization reactions, decomposition reactions, and complexation reactions.

### 4.1. Dissolution Effect

The particle size and cell wall structure of CHMs during the crushing process can impact the dissolution efficiency of saponin components, thereby affecting the extraction of saponins.

#### 4.1.1. Dissolution Effect Due to Particle Size

After the crushing and subsequent refinement of the CHMs, the specific surface area and effective contact surface area between the CHMs and the solvent are augmented, thereby enhancing the dissolution rate of saponin components [51]. However, when the particle size of the CHMs is refined to a certain degree, the specific surface area of the raw material particles increases significantly, resulting in a significant increase in surface energy by several orders of magnitude. This phenomenon is known as the “surface effect”. The excessive fineness of the CHMs particles can lead to the absorption of air and the generation of electrical charges, which can result in particle agglomeration and the formation of semi-stable ultrafine powder copolymers. Consequently, this phenomenon can lead to a reduction in the dissolution rate of active ingredients and the extraction rate of saponins [52,53,94]. The aforementioned studies indicate that the effective contact area between CHMs and the solvent can be altered by subjecting it to different particle size crushing processes. At the optimal particle size, the contact area with solvents is maximized while surface effects are minimized, thereby maximizing the extraction yield of saponins.

#### 4.1.2. Dissolution Effects Due to Wall-Breaking

**Figure 2:**
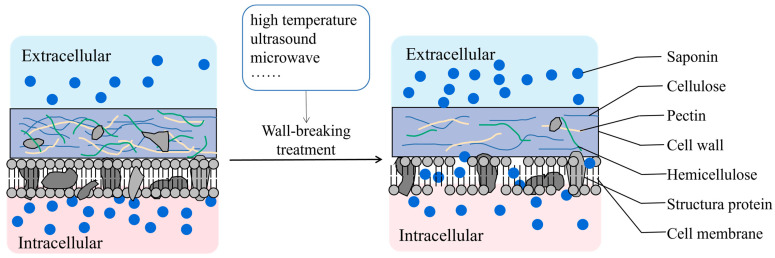
The dissolution of saponins increased after cell wall-breaking treatment.

The plant cell wall is a complex structure comprising numerous polysaccharides—such as cellulose, hemicellulose, and pectin—along with minor quantities of structural proteins and aromatic compounds. These components are held together by hydrogen and covalent bonds, which inhibit the dissolution of intracellular saponins [95]. The application of high-temperature, ultrasound, and microwave treatments during processing can increase the energy within plant tissues and promote cell wall rupture by exploiting the principles of mechanical effects and radiant heating (Figure 2) [96,97]. Polysaccharide-degrading enzymes found in plant cell walls, such as cellulase, hemicellulase, and pectinase, can also disrupt the cell wall structure through enzymatic hydrolysis, thereby accelerating the dissolution of saponins. Cellulase comprises endoglucanase, exoglucanase, and β-glucosidase. Initially, endoglucanase targets the amorphous region of cellulose, generating the free ends required for exoglucanase. Subsequently, exoglucanase continues to act on the reducing and non-reducing ends of cellulose chains to produce short-chain oligosaccharides such as cellobiose and cellotriose. Finally, β-glucosidase hydrolyzes the short-chain oligosaccharides to produce glucose, thereby disrupting the structure of the cell wall [98,99]. The aforementioned studies have demonstrated that heating, ultrasound, microwave, and enzymatic treatment of CHMs can disrupt the cell wall structure, facilitating the easier passage of intracellular saponin constituents through the cell wall and increasing their extraction rate.

### 4.2. Hydrolysis Reaction

Saponins can be hydrolyzed during processing by deglycosylation to secondary saponins or sapogenins. This hydrolysis can be divided into two types based on the presence or absence of enzymatic activity: enzymatic hydrolysis and non-enzymatic hydrolysis.

#### 4.2.1. Enzyme Hydrolysis

**Figure 3:**
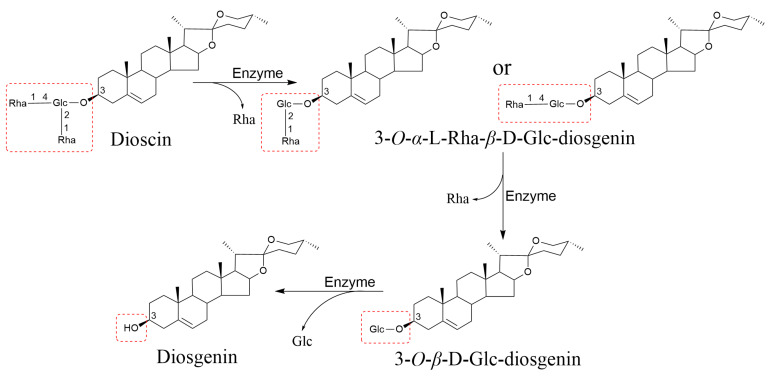
Dioscin-glycosidase hydrolysis pathway of dioscin.

During the processing of CHMs, enzymatic activity catalyzes the hydrolysis of saponins, leading to the formation of secondary saponins or sapogenins and consequently altering their content [100]. Ginsenosidase type I from the *Aspergillus niger* g.848 strain was used to deglycosylate ginsenosides Rb1, Rc, Rb2, and Rd from ginseng folium protopanaxadiol type ginsenosides, resulting in the production of the rare ginsenosides C-K [101]. The dioscin glycosidase produced by the strain Absidia sp. d38 can hydrolyze 3-*O*-*α*-L-(1→4)-Rha or 3-*O*-*α*-L-(1→2)-Rha from dioscin to produce 3-*O*-*α*-L-Rha-*β*-D-Glc-diosgenin. It also facilitates the rapid hydrolysis of additional α-L-Rha units and subsequently cleaves the *β*-D-Glc to form diosgenin (Figure 3) [102]. Enzymes such as glucosidases have specificity and can break different glucosidic bonds, leading to the hydrolysis of saponins and the removal of glycosyl groups. This results in changes in the structure and content of the saponins, which can have implications for their biological activity and potential applications. The sources of saponin-hydrolyzing enzymes during processing include enzymes released from the wall breaking of the herbs themselves and exogenous enzymes. It is therefore important to adequately control the release or activity of these enzymes in the raw materials during the extraction process to minimize saponin loss due to enzymatic hydrolysis.

#### 4.2.2. Non-Enzymatic Hydrolysis

In the processing of CHMs, high temperatures, acids, or alkalis can cause enzyme denaturation and inactivation. However, under acidic or alkaline conditions, saponins can be hydrolyzed to generate secondary saponins or sapogenins. After alkaline hydrolysis of *Astragali Radix*, the acetyl group (-OAc) on the glycosyl group in cycloastragenol saponin is replaced with hydroxyl group (-OH), resulting in the deacetylation and conversion to astragaloside A [103]. In *Ginseng Radix et Rhizoma* flowers, the glycosyl group in polar ginsenosides can undergo gradual hydrolysis to produce non-polar ginsenosides as the roasting temperature or roasting time increases [33]. Under acidic conditions, saikosaponin C can undergo ring ether opening and hydroxyl elimination at the C-13 position to generate saikosaponin H with a heterocyclic dienophile structure. This process is accompanied by a deglycosylation reaction at the C-3 position, resulting in the formation of de-rhamnose-saikosaponin C and de-rhamnose-saikosaponin H [104]. The mentioned studies indicate that saponins can undergo hydrolysis in the absence of enzymes, particularly at high temperatures or under acidic or alkaline conditions. This process leads to the production of secondary saponins or sapogenins and results in changes in the structure and content of the saponins.

### 4.3. Isomerization Reaction

**Figure 4:**
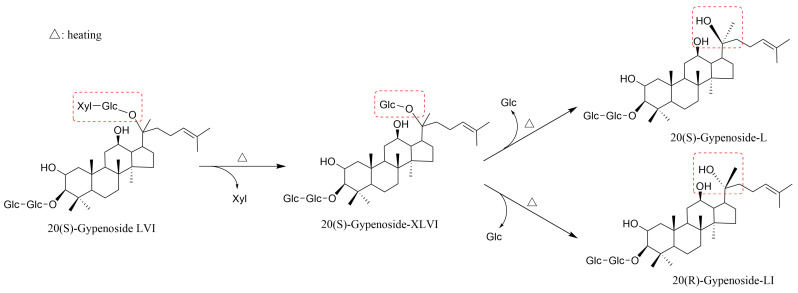
Conversion pathway of gypenosides during heat treatment.

Isomerization reaction refers to the process of converting an ingredient into its isomer. Saponins can indeed undergo isomerization reactions during processing, leading to changes in their structure and subsequently altering their pharmacological activity. During the thermal processing of ginseng, some ginsenosides undergo hydrolysis and isomerization, leading to the conversion of S-configurations and R-configurations secondary glycosides. In particular, ginsenoside Rb2 can be removed from pyranose at the C-20 position to form ginsenoside Rd. Subsequently, the glucose can be further removed to undergo an SN1 isomerization reaction, resulting in a transformation into 20 (S)-ginsenoside Rg3 and 20 (R)-ginsenoside Rg3 [105]. During hot processing, ginsenoside Re can undergo chemical transformations, which result in the production of 20 (S)-ginsenoside Rg2 and 20 (R)-ginsenoside Rg2 by removing the glucose at the C-20 position. Additionally, 20 (R)-ginsenoside Rh1 and 20 (S)-ginsenoside Rh1 can also be yielded by removal of the rhamnose at the C-3 position [33]. After the heat treatment of *Gynostemma pentaphyllum (Thunb.) Makino*, 20 (S)-gypenoside LVI is first converted by removing one glycosyl group to produce 20 (S)-gypenoside XLVI, and then by removing another glycosyl group to produce 20 (S)-gypenoside-L and 20 (R)-gypenoside-LI (Figure 4) [106]. The above studies have shown that during processing, saponins undergo isomerization to produce saponins with R and S configurations, altering both the content and pharmacological activity of the saponins.

### 4.4. Decomposition Reaction

**Figure 5:**
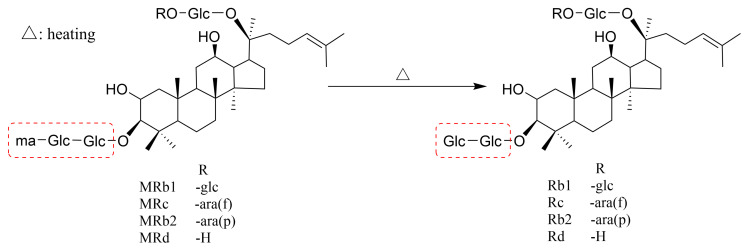
Thermal conversion pathway of malonyl ginsenosides.

**Figure 6:**
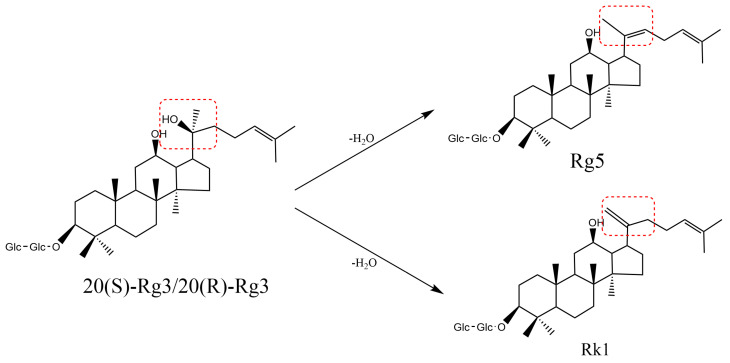
Dehydration condensation pathway of ginsenoside Rg3.

A degradation reaction is the reaction of a compound under certain conditions to break down into two or more compounds. The glycosides of saponins can be hydrolyzed to secondary saponins or modified saponins under certain conditions such as acidic or basic environments, enzymatic action, or high temperatures. When *Glycyrrhizae Radix et Rhizoma* is processed with high-temperature honey roasting, glycyrrhizic acid can undergo deglycosylation to form 18-β-glycyrrhetinic acid [107]. Under specific conditions such as acidic, alkaline, or high-temperature environments, the acyl bond of malonyl ginsenosides can undergo hydrolysis to remove malonic acid and produce other rare saponins (Figure 5) [36,44]. The hydroxyl group at the C-20 position of ginsenoside Rg2 can undergo dehydration condensation, resulting in the formation of ginsenoside F4 and ginsenoside Rg6 [33]. The hydroxyl group of ginsenoside Rg3 can undergo dehydration and condensation to form a double bond, resulting in the production of ginsenosides Rg5 and Rk1 at C-20 under heating conditions (Figure 6) [17]. The above studies have shown that the removal of glycosyl, hydroxyl, and malonyl groups from the saponin structure during processing produces new compounds which, in turn, affect the saponin content.

### 4.5. Complexation Reaction

Saponins contain hydroxyl groups, carbonyl groups, and carboxyl groups, which can form coordination compounds with inorganic metal ions through covalent bonds [108,109]. The formation of coordination compounds between *Chenopodium quinoa Willd.* saponins and silver can indeed enhance its antibacterial activity [110]. Platycodin D has been found to form coordination compounds with the metals ruthenium and iridium, which exhibit potent antitumor activity against SGC-7901 tumor cells [111]. The aforementioned studies indicate that saponins are capable of forming coordination complexes with metal ions within the storage and processing environments of CHMs. This interaction may alter the spatial configuration of the saponins, thereby potentially modifying the pharmacological activities of the medicinal material and its derivatives.

## 5. Summary and Prospects

CHMs undergo various storage conditions and complex processing from their origin to the final product, all of which may affect the stability of the saponin components they contain. This paper provides a comprehensive review of the research literature on the effects of storage and processing on the saponin components in CHMs. It systematically analyzes how different storage and processing conditions affect the saponin contents in CHMs. Ideally, the best storage conditions include low temperature, low humidity, low oxygen levels, and protection from light. However, in practical production and storage scenarios, due to cost and space constraints, enterprises or hospitals often resort to storing CHMs at room temperature with low humidity and light protection. For more valuable and rarer CHMs, low-temperature refrigeration with light protection is commonly employed [112]. After CHMs is processed into decoction pieces, it is usually packaged in sealed plastic bags. This type of packaging helps to maintain the stability of the appearance, moisture content, and active ingredient levels of the medicinal materials, with changes in component levels being significantly less than those in bulk materials. However, the effectiveness of plastic packaging can vary depending on the raw materials used. It is therefore imperative to avoid a one-size-fits-all approach to plastic packaging when selecting packaging materials; rather, the characteristics of the medicines, storage requirements, and transport conditions should be considered to select the most appropriate plastic packaging [24,113].

Different processing methods should be selected based on the desired tissue characteristics and component structures of CHMs. Appropriate drying, grinding, and extraction methods must be chosen accordingly. Additionally, fresh processing methods and traditional processing techniques can significantly influence the saponin contents in CHMs. For example, the total saponin content of *Notoginseng Radix et Rhizoma* dried at 50 °C after fresh cutting was 7.24% higher than that of traditional whole root dried at 50 °C [31]. As of December 31, 2023, 24 provinces have issued relevant guidelines for CHMs primary processing (fresh-cut), excluding those provinces that are still in the consultation phase. Fresh processing effectively prevents the loss of active components caused by “secondary processing” and shortens the production cycle [114]. During the processing, temperature has a significant impact on the saponin contents. pH and enzymes can lead to the hydrolysis of saponins, while metal ions may form complex reactions with saponins. Therefore, it is essential to control the relevant conditions during production. For instance, pre-treatments such as steaming or blanching in CHMs can deactivate enzymes, ensuring the retention of active components [115]; The extraction of monomeric saponins can be achieved by adding appropriate enzymes to maximize conversion yield [87]. In the process of decocting and extracting CHMs, it is advisable to avoid using iron or other metal utensils. The effects of storage and processing on the saponin components in CHMs involve various mechanisms, including dissolution effects, hydrolysis reactions, isomerization reactions, decomposition reactions, and complexation reactions. These processes can affect the number, configuration, and structure of the sugar moieties in the saponins, ultimately leading to changes in their content and pharmacological activity. Therefore, during the production and processing of CHMs, it is essential to choose appropriate storage and processing techniques based on a thorough assessment of the cost and benefit. This ensures the stability of the target saponin components during storage and processing, thereby achieving high-quality production of CHMs products.

Currently, there is limited research, both nationally and internationally, on the changes in saponin contents in CHMs during storage. Most of the studies focusing on changes in saponin content during processing have primarily focused on widely used herbs such as *Ginseng Radix et Rhizoma*, *Notoginseng Radix et Rhizoma*, and *Polygonati Rhizoma*. Improving the stability and bioavailability of saponins is a key technological challenge in the production process of saponin-rich CHMs. However, there are still several issues that need to be resolved: (1) The mechanisms underlying changes in saponin content during storage and processing have primarily focused on hydrolysis reactions, and other mechanisms need further investigation. (2) There is a need to strengthen the research on the effects of different storage conditions on saponin components in traditional Chinese medicine. This will help to establish optimal storage conditions for saponin-rich medicinal materials and prevent losses of saponin during storage. (3) Research should be intensified on the effects of various processing conditions on non-mainstream medicinal materials. Investigating the impacts of different drying, grinding, and extraction methods on the yield of saponin components will provide a scientific basis for the high-quality production and processing of saponin-containing CHMs.

## Figures and Tables

**Table 1:** The advantages and disadvantages of different extraction methods for saponins in Chinese herbal medicines (CHMs).

Method	Advantage	Disadvantage	Reference
Maceration method	Simple process, low cost and avoid the influence of temperature.	Large amount of solvent, Long extraction time and low efficiency.	[50]
Reflux method	Simple process, low cost and shorter extraction time.	Low safety factor and high temperature is easy to cause the loss of saponins.	[73]
Ultrasound-assisted method	Simple operation, higher extraction yield and eco-friendly.	Louder noise, mostly used in laboratory production, and industrial mass production is difficult.	[60,61]
Microwave-assisted method	Shorter extraction time, higher extraction yield and eco-friendly.	The actual operation is difficult to control, the generated energy is large and with a risk of triggering bumping.	[64,74]
Enzyme-assisted method	Simple operation, the shorter extraction time and higher extraction yield.	The appropriate enzyme needs to be selected, and experimental conditions are highly demanding.	[75,76]
Supercritical fluid extraction method	Higher extraction yield, resistance to oxidation and no solvent residue.	Higher investment costs, complex operation, and the extraction of saponins often requires the addition of entraining agents, such as water, methanol, ethanol, acetone, etc.	[69,77]

## Data Availability

Not applicable.

## References

[1] Zhi D., Zhu P.F., Hui L., Li X.C., Zhu Y.Y., Liu Y., Shi X.L., Chen W.D., Liu Y.P., Zhao Y.L. (2022). Discovery of potent immune-modulating molecule taccaoside A against cancers from structures-active relationships of natural steroidal saponins. Phytomedicine.

[2] Xu X.H., Li T., Fong C.M.V., Chen X.P., Chen X.J., Wang Y.T., Huang M.Q., Lu J.J. (2016). Saponins from Chinese Medicines as Anticancer Agents. Molecules.

[3] Zhu M.T., Sun Y.P., Bai H.D., Wang Y.M., Yang B.Y., Wang Q.H., Kuang H.X. (2023). Effects of saponins from Chinese herbal medicines on signal transduction pathways in cancer: A review. Front. Pharmacol..

[4] Yao L., Lu J., Wang J., Gao W.Y. (2020). Advances in biosynthesis of triterpenoid saponins in medicinal plants. Chin. J. Nat. Med..

[5] Passos F., Araújo-Filho H.G., Monteiro B.S., Shanmugam S., Araújo A., Almeida J., Thangaraj P., Júnior L., Quintans J. (2022). Anti-inflammatory and modulatory effects of steroidal saponins and sapogenins on cytokines: A review of pre-clinical research. Phytomedicine.

[6] Liu J., Xu Y.R., Yang J.J., Wang W.Z., Zhang J.Q., Zhang R.M., Meng Q.G. (2017). Discovery, semisynthesis, biological activities, and metabolism of ocotillol-type saponins. J. Ginseng Res..

[7] Xu C.F., Xia B.H., Zhang Z.M., Lin Y., Li C., Lin L.M. (2023). Research progress in steroidal saponins from the genus Polygonatum: Chemical components, biosynthetic pathways and pharmacological effects. Phytochemistry.

[8] Liang Y.T., Chen B.Q., Liang D., Quan X.X., Gu R.L., Meng Z.Y., Gan H., Wu Z.N., Sun Y.B., Liu S.C. (2023). Pharmacological Effects of Astragaloside IV: A Review. Molecules.

[9] Singh B., Singh J.P., Singh N., Kaur A. (2017). Saponins in pulses and their health promoting activities: A review. Food Chem..

[10] Jia A., Yang X.H., Zou B., Li J., Wang Y.F., Ma R.X., Li J., Yao Y. (2022). Saikosaponins: A Review of Structures and Pharmacological Activities. Nat. Prod. Commun..

[11] Fan W.X., Fan L.H., Wang Z.Y., Mei Y.Q., Liu L.C., Li L.N., Yang L., Wang Z.T. (2024). Rare ginsenosides: A unique perspective of ginseng research. J. Adv. Res..

[12] Qi L.W., Wang C.Z., Yuan C.S. (2010). American ginseng: Potential structure–function relationship in cancer chemoprevention. Biochem. Pharmacol..

[13] Li Y., Zhang P., Yu R., Chen R.Z., Si J.P., Zhang X.F. (2021). Effects of Different Storage Conditions on Edible Quality and Antioxidant Activity of *Polygonatum cyrtonema* Flowers. China J. Chin. Mater. Medica.

[14] Ma P., Li L.Y., Zhang Y. (2014). Impact of storage conditions and time on herb of *Lonicera macranthoides*. China J. Chin. Mater. Medica.

[15] Zhang L.W., Qiu H.L., Yuan S., Guo M.Z., Guo Z.Y., Yu L.J. (2018). Revelation of mechanism for aqueous saponins content decrease during storage of *Dioscorea zingiberensis* C. H. Wright tubers: An essential prerequisite to ensure clean production of diosgenin. Ind. Crop. Prod..

[16] Lee S.H., Kim K.M., Kim D., Han G.D. (2017). Changes in ginsenoside patterns of red ginseng extracts according to manufacturing and storage conditions. Food Sci. Biotechnol..

[17] Sun H. (2021). Study on Preparation Artwork and Anti-Cervical Cancer Activity Ofheat-Processed Hydrolysate of Ginsenosides Rb1. Master’s Thesis.

[18] Shi P.P. (2023). Preparation of Ginsenoside Rg3 by Biotransformation Ginsenoside Rb1 in Deep Eutectic Solvent. Master’s Thesis.

[19] Hwang C.R., Lee S.H., Jang G.Y., Hwang I.G., Kim H.Y., Woo K.S., Lee J., Jeong H.S. (2014). Changes in ginsenoside compositions and antioxidant activities of hydroponic-cultured ginseng roots and leaves with heating temperature. J. Ginseng Res..

[20] Zhu D.S., Zhang H. (2004). Effects of Different Temperature, Humidity and Packing Conditions on Storage of Dehydrated American Ginseng. Dry. Technol. Equip..

[21] Han X.T., Tang Y.N., Liu Z.Q., Bao L.Y., Han S., Liu K.Y., Wang H.Y., Guo X.G., Li W.F., Du H. (2022). Influence of different storage conditions on quality of *Codonopsis Radix* pieces. Chin. Tradit. Herb. Drugs.

[22] Zheng Y.F., Cai J.H., Tan Y.Q., Wei F., Wei H.J. (2023). Study on the Extraction and Stability of Saponin from Star Anise. China Condiment.

[23] Zhen L.H., Lu C.J., Zhou Z.K., Cao H., Wu S.Z. (2012). Determination of 5 saponins in total saponins of *Panax Notoginseng* by HPLC and study on their stability. Clin. J. Tradit. Chin. Med..

[24] Silpa S.G., Smitha G.R., Ranjitha K. (2021). Drying and Packaging Methods Impact the Bacoside Profile and Microbiological Quality of Brahmi Herb (*Bacopa monnieri* L.) During Storage. Ind. Crop. Prod..

[25] Yu F., Wan N., Li Y.H., Wang X.C., Wu Z.F., Liu Z.F., Yang M. (2021). Analysis on the Change Rule and Mechanism of Physicochemical Properties of Chinese Medicinal Materials During Drying. Chin. Tradit. Herb. Drugs.

[26] Nurhaslina C.R., Sharlien A.B., Mustapa A.N. (2022). Review on drying methods for herbal plants. Mater. Today Proc..

[27] Li L.S., Chen L.P., Pan D.J., Zhu Y., Huang R.S., Chen J., Ye C.Y., Yao S.C. (2024). Evaluation of different drying methods on the quality of *Cinnamomum cassia* barks by analytic hierarchy process method. Heliyon.

[28] Dong L.H., Kuang Y.H., Fan D.D., Jiang T., Chen L.M., Zhang D., Zhu J.J., Wang Z.M., Wang D.Q., Li C.Y. (2018). Comparison of saponins from *Gynostemma pentaphyllum* leaves prepared by different processing methods. China J. Chin. Mater. Medica.

[29] Yazici M., Kose R. (2024). Energy, exergy and economic investigation of novel hybrid dryer, indirect solar dryer and traditional shade drying. Therm. Sci. Eng. Prog..

[30] Fan T.C., Du Z.Y., Li J., Wen Y.L., Liu L.T., Liu Y.N. (2021). Effects of Different Drying Methods on Chemical Components of Traditional Chinese Medicine: A Review. Mod. Chin. Med..

[31] Liu Y., Chen J.F., Xu N., Lin W.G., Liu Y.M., Chen M.L., Liu D.H. (2019). Effects of fresh-cut on drying rate and quality of *Panax notoginseng*. China J. Chin. Mater. Medica.

[32] Xu M.D., Zhang X.J., Liu X.K., Gong J.Y., Yu P., Xie X.Y., Cai G.R. (2023). Quality evaluation of *Panax ginseng* decoction slices based on fresh cutting and traditional processing technology. Chin. Tradit. Herb. Drugs.

[33] Li X. (2018). Effects of Thermal Processing on Phenolic Components, Antioxidant Activities and Saponins Conversion Mechanisms of *Panax ginseng* C.A. Meyer Flowers and Pulps. Master’s Thesis.

[34] Ning X.F., Feng Y.L., Gong Y.J., Chen Y.L., Qin J.W., Wang D.Y. (2019). Drying features of microwave and far-infrared combination drying on white ginseng slices. Food Sci. Biotechnol..

[35] Li T., Yan Y.T., Han L.L., Li M.K., Liu S.Y., Zhou X.Y., Lee J., Li X.M., Zhao Y.Q. (2024). Ginseng fruit rare saponins (GFRS), a promising anti-wrinkle agent: Evidence of its antioxidant effect and its capacity to prevent matrix metalloproteinase (MMPs) expression in vitro and in vivo. Ind. Crop. Prod..

[36] Zhang D.L., Li M.Y., Wang D.S., Wen X., Li J.X., Liu Z. (2023). Effects of Different Heating Methods on Degradation of Malonyl ginsenosides and Changes in Antioxidant Activity. J. Jilin Agric. Univ..

[37] Liu Z., Xia J., Wang C.Z., Zhang J.Q., Ruan C.C., Sun G.Z., Yuan C.S. (2016). Remarkable Impact of Acidic Ginsenosides and Organic Acids on Ginsenoside Transformation from Fresh Ginseng to Red Ginseng. J. Agric. Food. Chem..

[38] Awang D.V.C. (2000). The Neglected Ginsenosides of North American Ginseng (*Panax quinquefolius* L.). J. Herbs Spices Med. Plants.

[39] Li Q.H., Duan L.H., Lv D., Cui X.M. (2023). Optimization of vacuum freeze-drying process and comparison of anticoagulation effect in *Panacis Majoris Rhizoma*. J. Chin. Med. Mater..

[40] Heng Y.X., Zheng X.X., Yin Z.Y., Bian F.X., Liu D.D. (2018). The effects of different drying methods on the drying characteristics and quality of *Polygonatum odoratum*. Sci. Technol. Food Ind..

[41] Song L.H., Zhang R., Xu T.Y., Yu P. (2023). Effects of different drying methods and temperatures on the content of chemical components and antioxidant activity of Ginseng decoction pieces. Lishizhen Med. Mater. Medica Res..

[42] Luo Y., Li W.Q., Wan F.X., Huang X.P. (2020). Drying Characteristics of *Platycodon grandiflorum* Slice by Hot-Wind Based on Weibull Distribution Function. J. Agric. Sci. Technol..

[43] Zhang J., Ding B., Zhang H., Qi J.S., Shen Y.X., Zhou N., Chen X.H., Yang D.M. (2016). Effects of Different Drying Methods on Total Saponin Content and Antioxidant Capacity of *Paris polyphylla* var.yunnanensis. Chin. J. Inf. Tradit. Chin. Med..

[44] Li F., Li Q., Song D., Liu P.P., Lu C.N., Wang J., Jia L.Y., Lu J.C. (2015). Effect of different drying methods on ginsenosides in flower of *Panax ginseng* and *Panax quinquefolius*. Chin. Tradit. Herb. Drugs.

[45] Wang L.Y., Li C.H., Liu M.Z., Li K., Ye X.R., Wang Z.W., Fan L.T., Wang R.K., Zhao H.Y., Kan Z. (2024). Research status of drying technology and equipment of Chinese medicinal materials. Trans. Chin. Soc. Agric. Eng..

[46] Pei Y., Li Z., Song C., Li J., Song F.H., Zhu G.Y., Liu M.B. (2019). Effects of combined infrared and hot-air drying on ginsenosides and sensory properties of ginseng root slices (*Panax ginseng Meyer*). J. Food Process Preserv..

[47] Guo X.Y., Yang D.S., Ma C.H., Wang W.C., Huang J.M., Yin Y.S. (2012). The Influence of Different Drying Techniques for the Contents of Effective Ingredients in *Rhizoma Anemarrhenae*. Chin. J. Ration. Drug Use.

[48] Liu X.S., Qiu Z.F., Wang L.H., Chen Y. (2010). Quality evaluation of *Panax notoginseng* extract dried by different drying methods. Food Bioprod. Process..

[49] Popovich D.G., Hu C., Durance T.D., Kitts D.D. (2005). Retention of Ginsenosides in Dried Ginseng Root: Comparison of Drying Methods. J. Food Sci..

[50] Liu D., Yu Z.X., Sang M., Gu Y., Gao Y., Tang Y.F., Qian F. (2020). Analysis of problems related to the pre-mashing of Chinese medicine pieces. Lishizhen Med. Mater. Medica Res..

[51] Luo G., Chen L.T., Zhou J. (2011). Application of ultra-fine pulverization technology in Chinese materia medica. Drugs Clin..

[52] Li C.H. (2002). Study on Superfine Pulverization of Chinese Traditional Medcines and Dissolving-Out Characteristics of Effective Compositions. Ph.D. Thesis.

[53] Zhang J., Wang X.Q., Wang D.Q., Hou J.G. (2009). Effects of Superfine Comminution on Leaching of Ginsenosides in *Panax ginseng*. Food Sci..

[54] Islam M., Malakar S., Rao M.V., Kumar N., Sahu J.K. (2023). Recent advancement in ultrasound-assisted novel technologies for the extraction of bioactive compounds from herbal plants: A review. Food Sci. Biotechnol..

[55] Peng Y.Q., Liu S., Kang Z.P., Du C.Y., Liang Z.S. (2021). Study on Extraction Technology and Content Determination of Saikosaponin. Chem. Reag..

[56] Costa J.R., Tonon R.V., Cabral L., Gottschalk L., Pastrana L., Pintado M.E. (2020). Valorization of Agricultural Lignocellulosic Plant Byproducts through Enzymatic and Enzyme-Assisted Extraction of High-Value-Added Compounds: A Review. ACS Sustain. Chem. Eng..

[57] Zhao H.Y., Liu F.Q., Wang Y.P., Zhao N., Wu Y.Y., Wang X.Y. (2009). Comparative study of extraction process on the effective components of *Panax notoginseng*. J. Pharm. Pract. Serv..

[58] Feng H.Q., Xu X.F., Yang H.W., Shen B.Y., Hu J., Li C.Z. (2016). Comparative studies on different extraction process of saponin from quinoa seeds. Sci. Technol. Food Ind..

[59] Chen M., Liao Y., Wu Y., Li L. (2021). Comparative Study on Different Extraction Methods of Jujuboside A. Farm Prod. Process..

[60] Wen C.T., Zhang J.X., Zhang H.H., Dzah C.S., Zandile M., Duan Y.Q., Ma H.L., Luo X.P. (2018). Advances in ultrasound assisted extraction of bioactive compounds from cash crops—A review. Ultrason. Sonochem..

[61] Xue F., Li C.N., Li P.S., Liu Y.Y., Fan B.D., Bao H.Y., Xu T.H., Liu T.H. (2014). Application in Ultrasonic Extraction Chemical Constituents of Traditional Chinese Medicine. Chin. J. Exp. Tradit. Med. Formulae.

[62] Choudhary N., Chatterjee M., Kumar S., Singh G., Suttee A. (2021). Effect of conventional method and microwave assisted extraction on phytoconstituents of *Chenopodium album*. Mater. Today Proc..

[63] Dahmoune B., Houma-Bachari F., Chibane M., Akrour-Aissou C., Guégan J., Vives T., Jéhan P., Dahmoune F., Mouni L., Ferrières V. (2021). Microwave assisted extraction of bioactive saponins from the starfish *Echinaster sepositus*: Optimization by response surface methodology and comparison with ultrasound and conventional solvent extraction. Chem. Eng. Process..

[64] Fan Y., Li Z.M., Liu L., Xi J. (2020). Combination of liquid-phase pulsed discharge and ultrasonic for saponins extraction from lychee seeds. Ultrason. Sonochem..

[65] Yang J.J., He S.G., Zhao M.Q., Zhang X. (2016). Compared study on extraction of total gypenosides by different methods. Sci. Technol. Food.

[66] Shan H.J. (2022). Study on Extraction and Isolation of Saponins from Chenopodium quinoa and its Anti-Colon Cancer. Master’s Thesis.

[67] Wang M.J., Wang L.L., Du X.X., Zhang T.J., Gong S.X., Yang J.Y. (2012). Effects of different extracting methods on total saponins extraction efficacy from *Smilax china*. Chin. Tradit. Herb. Drugs.

[68] Li J.J., Fan S.W., Yu R.L. (2024). Analysis of active components and antioxidant activity of *Astragalus astragalus* by supercritical fluid extraction. J. Chin. Med. Mater..

[69] Dassoff E.S., Li Y.O. (2019). Mechanisms and effects of ultrasound-assisted supercritical CO_2_ extraction. Trends Food Sci. Technol..

[70] Li Z.J., Xu Y.B., Liu Y.W., Kong M.R., Ma K., Wang J.R., Wang H.G., Zhao Y.H. (2024). Ultrasonic pretreatment combined with microwave-assisted hydrodistillation for the situ extraction of essential oil from Pinus koraiensis seed scales induced by tea saponin: Functional activity, composition, thermal stability and material characterization. Ind. Crop. Prod..

[71] Yan Y.X., Fu Z.X., Wan J.F., Zhang Y.J., Gao J.L., Gao J.Q., Wang W. (2024). Enhancing the recovery of complex amino acids from excess sludge via low-intensity ultrasound-assisted enzymatic hydrolysis. Chem. Eng. J..

[72] Zhang H.L., Li X.P., Kang M., Li Z.R., Wang X.W., Jing X., Han J.J. (2023). Sustainable ultrasound-assisted extraction of Polygonatum sibiricum saponins using ionic strength-responsive natural deep eutectic solvents. Ultrason. Sonochem..

[73] Ou X.H., Wang X., Yang Y., Liu W., Jin H., Xiao Y.B., Liu D.H. (2012). Study on Extraction Conditions of *Panax notoginseng* Saponins by Orthogonal Experiment. Mod. Chin. Med..

[74] Qi H., He Y.N., Wang F., Wu J., Ci Z.M., Chen L.M., Xu R.C., Yang M., Lin J.Z., Han L. (2021). Microwave technology: A novel approach to the transformation of natural metabolites. Chin. Med..

[75] Ke S.T., Zhu Y.Y. (2021). Analysis on Extraction Technology of Effective Components of Chinese Herbal Medicine and Detection Method of Toxic Components. Mod. Chem. Res..

[76] Rafińska K., Wrona O., Krakowska-Sieprawska A., Walczak-Skierska J., Kiełbasa A., Rafiński Z., Pomastowski P., Kolankowski M., Buszewski B. (2022). Enzyme-assisted extraction of plant material—New functional aspects of the process on an example of *Medicago sativa* L.. Ind. Crop. Prod..

[77] Wrona O., Rafińska K., Możeński C., Buszewski B. (2017). Supercritical Fluid Extraction of Bioactive Compounds from Plant Materials. J. Aoac Int..

[78] Xing Y.B., Wang X.Y., Wang M.Y., Qi X., Cui C.B. (2024). Process Optimization of Total Saponins from Adventitious Roots of Ginseng and Their Antioxidant and Anti-fatigue Effects. Sci. Technol. Food Ind..

[79] Yuan L. (2015). Study on the Preparation of Total Saponins and Polysaccharides from *Polygonati rhizoma* and the Effect of Soil Moisture Content on Its Quality during Cultivation. Master’s Thesis.

[80] Xu X.F., Gao Y., Xu S.Y., Liu H., Xue X., Zhang Y., Zhang H., Liu M.N., Xiong H., Lin R.C. (2018). Remarkable impact of steam temperature on ginsenosides transformation from fresh ginseng to red ginseng. J. Ginseng Res..

[81] Li J., Chen L.N., Jiang H., Zhang Q., Zhang Y.P. (2014). Study on the Degradation of Saikosaponin a in Water Solution. Lishizhen Med. Mater. Medica Res..

[82] Shi D.Z., Wu F.L., Tan L.Y., Zhou B.S., Liu J.P., Li P.Y., Lai S.H. (2022). Optimization of preparation of ginsenoside Rg2 by alkali-hydrolyzed ginseng stem and leaf triol saponins by Box-Behnken Design-response surface method. J. Chin. Med. Mater..

[83] Zhang X., Song F.R., Liu Z.Q., Liu S.Y. (2006). Studies on the Stripping Regularity of Ginsenosides in Aqueous Solutions with Different pH Values by HPLC-ESI-MSn. Chem. J. Chin. Univ.-Chin..

[84] Zhang G.S., Feng C.H., Luo X.J., Su S.N., Hu P.Y., Wang Y.S. (2011). Optimization of Extraction Technology for Saikosaponins from Bupleurum. Chin. J. Exp. Tradit. Med. Formulae.

[85] Tan X.M. (2021). Study on the Furostanol Saponins from *Paris polyphylla* var. chinensis and their Conversion Mechanism during Drying Processing. Master’s Thesis.

[86] Wang L.J. (2019). Cloning and Catalytic Function of *Anemarrhenae rhizoma* Steroidal Saponin 26-O-β-Glucosidase Gene. Master’s Thesis.

[87] Xu T.Z., Wu M.R., Shao J.L., Chang Y., Zhu Y.T., Chen J.X., Chen L.Y., Chen Y.D., Yang H., Xia G.H. (2023). Preparation of rare protosaikosaponin A by enzymatic hydrolysis of saikosaponin B1. Chin. Tradit. Pat. Med..

[88] Mok I., Jung H., Kim H., Kim D. (2023). Biotransformation of ginsenosides from Korean wild-simulated ginseng (*Panax ginseng* C. A. Mey.) using the combination of high hydrostatic pressure, enzymatic hydrolysis, and sonication. Food Biosci..

[89] Wei X.Y., Guo X.N., Wei L.Z., Gong J.F., Leng X.X., Cai D.Y. (2020). Extraction and antioxidant activity of total saponins from *Chenopodium quinoa*. J. Tradit. Chin. Vet. Med..

[90] Chen Q.L., Hua Y., Wang C.L., Zhang N., Sun Y.N., Wang Z., Liu J.L. (2014). Analytical method to evaluate chelating capacity of constituents in decoction of Chinese materia medica with free iron ions. Chin. Tradit. Herb. Drugs.

[91] Cao B.Y., Xu S.Y., Li G.H., Wang Y., Yu H.S. (2024). Catalytic effect of metal ions on ginsenoside Rb1 in an ethanol system. Chem. Reag..

[92] Gui L., Xu L.Q., Song J.G., Yu H.S. (2020). Optimization of preparation process of rare ginsenosides Rk1 and Rg5 catalyzed by metal ions. J. Dalian Polytech. Univ..

[93] Yuan T., Liu C.Y., Xu L.Q., Song J.G., Yu H.S. (2019). Metal ions catalyzed conversion of ginsenoside Rg1 to Rh1. J. Dalian Polytech. Univ..

[94] Lucie K., Václav S., Gabriela Z., Tomáš Z. (2023). The effect of porosity and particle size on the kinetics of porous carbon xerogels surface oxidation. Carbon.

[95] Kubicek C.P., Starr T.L., Glass N.L. (2014). Plant cell wall-degrading enzymes and their secretion in plant-pathogenic fungi. Annu. Rev. Phytopathol..

[96] Hu W.W. (2021). The Mechanism of Manosonication-Assisted Extraction of Rhamnogalacturonan I (RG-I) Enriched Pectic Polysaccharides. Ph.D. Thesis.

[97] Yang Y., Zhang J.L., Zhou Q., Wang L., Huang W., Wang R.D. (2019). Effect of ultrasonic and ball-milling treatment on cell wall, nutrients, and antioxidant capacity of rose (*Rosa rugosa*) bee pollen, and identification of bioactive components. J. Sci. Food. Agric..

[98] Hao Q., Den Q.C., Zhou B., Chen Y.M., Zhou Q., Chen H.J., Den Z.Y., Chen Y.S. (2024). High efficiency enzymatic hydrolysis technique of polysaccharide in plant cell wall and its application in food processing. Food Sci..

[99] Thite V.S., Nerurkar A.S. (2020). Crude Xylanases and Pectinases from Bacillus spp. Along with Commercial Cellulase Formulate an Efficient Tailor-Made Cocktail for Sugarcane Bagasse Saccharification. Bioenergy Res..

[100] Li M., Huang Z., Zhang R., Zhou J. (2024). Review of probiotics, gut microorganisms, and their enzymes involved in the conversion of ginsenosides. Food Biosci..

[101] Song Y., Xu S., Yu H. (2024). Preparation of rare ginsenosides C-K by enzymatic transformation of ginseng-leaf protopanaxadiol type ginsenosides. Food Ferment. Ind..

[102] Fu Y.Y., Yu H.S., Tang S.H., Hu X.C., Wang Y.H., Liu B., Yu C.X., Jin F.X. (2010). New dioscin-glycosidase hydrolyzing multi-glycosides of dioscin from Absidia strain. J. Microbiol. Biotechnol..

[103] Zhang J.H., Zhou J., Song H.Q., Wu Z.L. (2010). Study on technology of improving astragaloside in Astragalus herb by alkaline hydrolysate. Lishizhen Med. Mater. Medica Res..

[104] Wang Z.H., Zhao M.Y., Tian L., Chen S.J., Jiao L.L., Liu S.Y., Zhao H.X., Xiu Y. (2023). Structure and pathway analysis of chemical transformation products of saiko-saponins c based on HPLC-Q Exactive-Orbitrap mass spectrometry. Chin. J. Anal. Lab..

[105] Kang K.S., Kim H.Y., Baek S.H., Yoo H.H., Park J.H., Yokozawa T., Ainstitute O.N.M., University O.T., Bcollege O.P., Seoul N.U. (2007). Study on the Hydroxyl Radical Scavenging Activity Changes of Ginseng and Ginsenoside-Rb2 by Heat Processing. Biol. Pharm. Bull..

[106] Duan Y., Yang J., Xie J.B., Xie P., Qi Y.S., Zhao M.T., Piao X.L. (2021). Simultaneous quantitative analysis of nine saponins in Gynostemma pentaphyllum before and after heat processing based on UPLC-Q-Trap-MS. China J. Chin. Mater. Medica.

[107] Sung M.W., Li P.C.H. (2004). Chemical analysis of raw, dry-roasted, and honey-roasted licorice by capillary electrophoresis. Electrophoresis.

[108] Wang Q., Shen J. (2021). Research progress of metal complexes in traditional Chinese medicine. J. North Pharm..

[109] Hassan E.A., Abou E.W., Abo-Elfadl M.T., Hassan M.L. (2021). New pectin derivatives with antimicrobial and emulsification properties via complexation with metal-terpyridines. Carbohydr. Polym..

[110] Jing J.J., Zhang R.Y., Du X., Du L.D., Liu Z.L., Lin B.J., Zhang F.X., Xue P. (2021). Synthesis and Synergistic Antibacterial Activity of Complexes of *Quinoa Saponins* with Silver Nanoparticles. Sci. Technol. Food Ind..

[111] Zeng C.C. (2018). The Study on Antitumor Activity of Platycodin D and Metal (Ruthenium, Iridium) Complexes. Master’s Thesis.

[112] Zhou W.J. (2017). Study on the Effects of Different Storage Environments and Packaging Materials on Quality of *Semen Arecae* and other Traditional Chinese Medicines. Master’s Thesis.

[113] He W.W., Hang D.D., Wei C.C., Lv R., Jin L. (2018). Research on the correlation between packaging and quality of Chinese medicinal materials. J. Chin. Med. Mater..

[114] Li P.Y., Zheng J.C., Yang H.B., Wang J.W., Zen Y., Du J., Wang J.Y. (2024). Policy Implementation Effects and Suggestions on Primary Processing (Fresh-cut) of Chinese Medicinal Materials. Mod. Chin. Med..

[115] Yang P. (2022). Study on Lily Bulb Drying Based on Blanching Pretreatment Process Optimization. Master’s Thesis.

